# Closing the birth registration gap for *Every newborn* facility birth: literature review and qualitative research

**DOI:** 10.1080/16549716.2023.2286073

**Published:** 2023-12-12

**Authors:** Masudah Paleker, Dorothy Boggs, Debra Jackson, Louise-Tina Day, Joy E. Lawn

**Affiliations:** a Maternal, Adolescent, Reproductive & Child Health Centre, London School of Hygiene & Tropical Medicine, London, UK; b Western Cape Government: Health, Cape Town, South Africa; c International Centre for Evidence in Disability, London School of Hygiene & Tropical Medicine, London, UK; d School of Public Health, University of the Western Cape, Cape Town, South Africa

**Keywords:** CRVS, birth registration, LMIC, facility-based, newborn

## Abstract

**Background:**

Birth registration is vital to provide legal identity and access to essential services. Worldwide, approximately 166 million children under five years (just under 25%) are unregistered, yet >80% of all births occur in health facilities in most low- and middle-income countries (LMIC).

**Objectives:**

This study, conducted in association with UNICEF, aims to review facility-based birth registration initiatives, and provide recommendations to close the gap between facility birth and birth registration rates in LMIC.

**Methods:**

A literature review covering published and grey literature was conducted to identify facility-based initiatives to increase birth registration rates. Semi-structured in-depth interviews were conducted by audio-call with six key global stakeholders to identify additional initiatives, and further insights for barriers and enablers to close the gap.

**Results:**

Academic databases and grey literature search yielded 21 studies meeting pre-specified inclusion criteria. Nine barriers preventing birth registration were identified and grouped into three themes: health system, governmental, and societal barriers. Facility-based birth registration initiatives resulted in an increase in birth registration rates. Importantly, health promotion within communities also increased demand for birth registration. In-depth interview respondents provided further detail and supported data found in literature review. Synthesis of the literature and stakeholder interviews noted enablers including inter-sectoral collaboration between health sector and civil registration ministries e.g., placing civil registration offices in health facilities or allowing medical doctors to act as registrars.

**Conclusion:**

Facility-based birth registration initiatives can increase birth registration rates in LMIC. Initiatives need to address both supply and demand side of birth registration to improve facility-based birth registration rates. A multi-sectoral approach within governments, and alignment with multiple stakeholders is vital.

## Introduction

Identity registration is crucial in providing children with important rights including access to healthcare and education. United Nations Children’s Fund (UNICEF)’s progress report on birth registration estimated that approximately 166 million children under five years of age, just under 25%, were unregistered [1,2]. Without a formal birth registration an individual does not legally exist [3]. According to the World Health Organization (WHO) approximately 99% of unregistered births occur in low- or middle-income countries (LMICs) [4] with nearly 80% within southern Asia and sub-Saharan Africa [5]. One in three children under five globally do not possess proof of birth registration in the form of a birth certificate [1]. The target for Sustainable Development Goal (SDG) 16.9 is to have a legal identity for all globally by the year 2030 [6]. UNICEF is supporting this by tracking the progress towards universal birth registration [1]. Barriers to birth registration have been described as either supply-related, demand-related, or a combination, and contribute to the delays or non-registration of children at birth [1,5]. Common barriers include financial cost of registration, distance to registration centres, and lack of awareness and knowledge about birth registration, all of which could be addressed through facility-based registration at the time of birth [7,8].

The Every Newborn Action Plan (ENAP) was endorsed by the World Health Assembly in 2014, is led by the WHO and UNICEF, and provides a framework based on evidence to end preventable neonatal deaths and stillbirths by 2035 [9]. ENAP’s strategic objective 5 is to ‘count every newborn through measurement, programme tracking and accountability’ [10]. Registration of births allow for easier counting and tracking which could ultimately be used to improve health outcomes. Almost a quarter of countries worldwide lack quality data to monitor Civil Registration and Vital Statistics (CRVS) coverage adequately [1], which makes tracking SDG 16.9 that much more challenging. CRVS systems are weaker in countries with higher burden of stillborn and newborn deaths [11]. Offering birth registration within facilities at the time of birth provides an opportunity to enhance data quality [9], which would allow for broader statistics to be produced regionally and nationally for large-scale reporting and planning, and can also be used by the health system directly for improved and targeted health care provision [12]. There are limited reviews on birth registration initiatives [13], and none focusing specifically on facility-based birth registration initiatives in LMIC.

The global gap between newborns that are delivered in facilities and are registered is 4.3%, while this gap in the least developed countries and sub-Saharan Africa are higher at 12.8% and 14.2% respectively [14]. There is an opportunity for rapid progress by closing the gap between global facility birth rates, but there is a lack of information on facility-based birth registration initiatives in LMIC and the barriers parents face to secure birth registration for their child.

### Aims and objectives

This study aims to assess facility-based birth registration initiatives and innovations in LMICs to provide recommendations to close the gap between facility births and birth registration rates.

Specific objectives are to:

1. Identify facility-based birth registration barriers, enablers and initiatives in LMICs through a literature review.
2. Undertake in-depth semi-structured interviews with key global birth registration stakeholders to identify current global initiatives, and explore enablers, barriers and gaps for facility-based birth registration in LMICs.
3. Synthesise barriers and enablers from the literature and the in-depth interviews in order to inform recommendations.

## Methods

### Literature review to identify facility-based birth registration initiatives and barriers in LMICs

**Figure 1.**
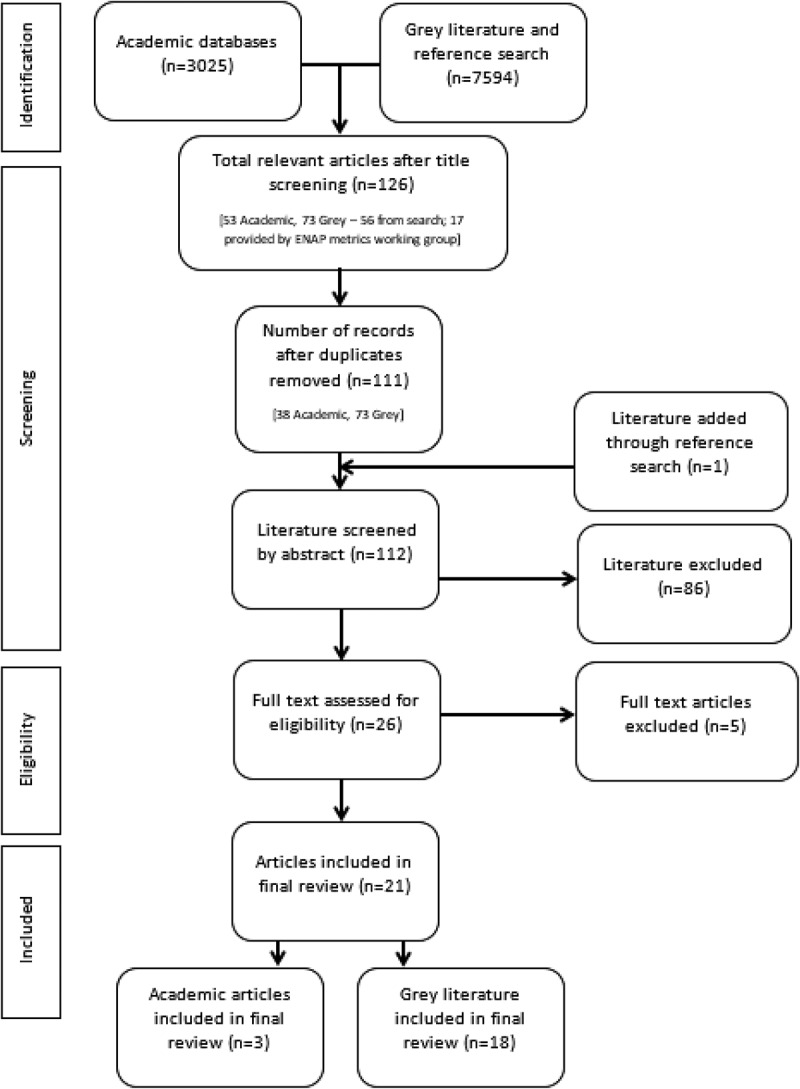
Prisma (28) flow chart diagram presenting literature review search results.

A search was done on both academic and grey literature with no limits set for time, language or type of article. Search terms included ‘health facility’, ‘hospital’, ‘clinic’, ‘birth registration’, ‘birth certificate’, ‘Civil Registration and Vital Statistics’ and ‘CRVS’ and was done using five databases: CINAHL Plus, Cochrane Library, Global Health, MEDLINE, PubMed and Web of Science. Grey literature, articles, studies and reports were identified through searching on selected organisations websites (UNICEF, WHO, Plan International), using Google search engine and in discussion with experts. The detailed search strategy can be found in Supplemental online material 1. Reference lists of studies that met inclusion criteria were searched which yielded one additional academic journal article which was included. The Prisma tool and checklist was used to refine the search (Figure 1).

Duplicates were eliminated and articles were included if they met all inclusion criteria: the initiative or innovation had to be health facility-based in an LMIC; with the aim of improving birth registration rates; either completed or ongoing with interim results; and reported on each of the three outcomes of interest, i.e. birth registration rates after initiation of an intervention, barriers to accessing and utilizing birth registration services and recommendations to improve these services. Articles were excluded if they were published in a language other than English and not officially translated by the authors.

An adapted version of the Cochrane Data Collection form [15] was used for data extraction including stakeholder information, region, methodology, intervention and outcomes of interest (Supplemental online material 2). Results of the literature review were documented using a narrative synthesis method.

### Semi-structured interviews with birth registration stakeholders to identify initiatives, enablers, barriers and gaps

The COREQ guidelines [16] were used to report on the qualitative research. Following discussions with members of the ENAP metrics working group (DB, JL, LTD), key stakeholders (*n* = 19) working to improve facility-based birth registration at various global organisations, academic institutions and non-profit foundations within different sectors were identified using purposive and subsequent snowball sampling. A letter of request for an interview was sent by email (Supplemental online material 3), with one follow-up email sent to non-responders.

An interview guide was developed, informed by the literature and reviewed by aforementioned ENAP metrics working group members to meet the objectives. Participants were given an information sheet, interview guide and a detailed consent form (Supplemental online material 4 and 5) in English at least three days prior to the interview. One female researcher (MP) with no prior relationship to participants conducted all interviews in English via audio call ranging from 20 to 45 minutes. At the time of interview, the researcher was qualified with an MBChB and an MSc Public Health candidate with training in social research methodology. Interviews were audio-recorded, transcribed, anonymized and stored on a secure server. NVivo10 software was used for data management by the interviewer.

Data were coded into pre-determined themes that were informed by the literature review, with additional themes added during analysis. The results of each theme were compared, and data were synthesised into narrative form to inform the results and recommendations. Recommendations were based on both the literature review and interviews and were presented using the same themes as the results.

## Results

### Literature review

A total of 3025 records between 2009 and 2017 were identified, and 21 articles met inclusion criteria. See Figure 1 for the Prisma flow chart search diagram.

The 21 publications included peer-reviewed articles (*n* = 3), progress reports (*n* = 8), policy reports (*n* = 2), website articles (*n* = 2), case studies (*n* = 3), working papers (*n* = 2), and a situational analysis (*n* = 1). Geographies represented included: 10 articles were based in Africa, nine in Southern Asia and Oceania, one in Latin America and the Caribbean, and three South America. Included in the above, one progress report focused on birth registration in three different countries namely Brazil, The Gambia and Bangladesh.

Among three peer-reviewed articles, two [17,18] used mixed methods and one [19] was a before and after intervention study. Kaneko et al [18], aimed to improve birth registration rates in Burundi through implementation of a family held Mother and Child Handbook with a specific section for birth registration. The intervention was assessed by means of a questionnaire pre- and post-distribution of the Handbooks. Secondary data on birth registration rates in the region were obtained from the national health management system and compared before and after the intervention. Kumar et al. [17] conducted a mixed-methods study using process evaluation and secondary data analysis. They reviewed the effects of a new policy in Haryana, India which shifted the responsibility of birth registration from the police to health facilities. Mony et al. [19] reviewed the effects of a nongovernmental organisation initiative called Strengthening Local Vital Event Registration at subdistrict level in Southern India with the aim of creating a direct link for civil registration data between hospitals and government in order to improve birth registration rates. They also conducted demand-side interventions where project staff visited pre-determined households in the region to promote the importance and need for birth registration. Birth registration rates were measured before and after the intervention.

The grey literature discussed barriers to birth registration within a defined geographical area and improved birth registration rates after initiation of interventions put in place by governments or NGOs. The articles provided recommendations for steps to maintain and further improve birth registration within that specific country or region. Secondary data, including census and other governmental statistics, were used to inform the outcome measures.

#### Facility-based birth registration initiatives

**Figure 2.**
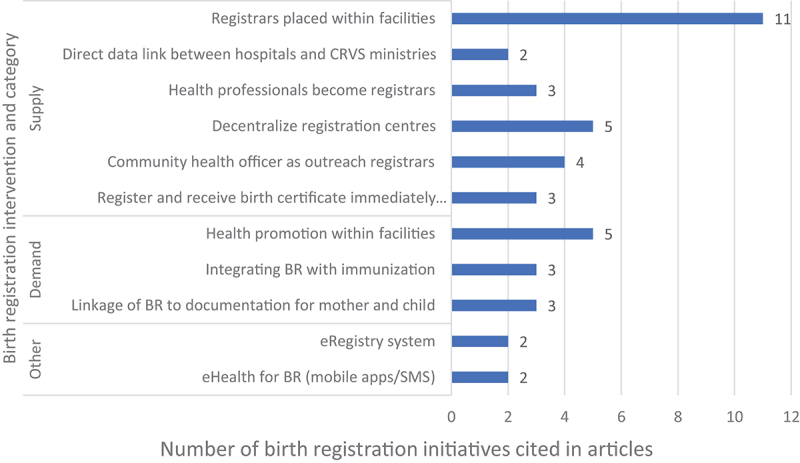
Birth registration interventions (*n* = 43) used as found in the literature review articles (*n* = 21).

Most articles (*n* = 11) described introduction of registrars employed by the national civil registry into health facilities, instead of their usual central placement, as an intervention to improve birth registration rates. Among included articles, five used health promotion within health facilities to increase awareness and educate parents on the importance of birth registration. The health promotion campaigns included health education at routine service points such as antenatal visits and to pregnant mothers on admission for delivery. Integrating birth registration with other health contact points such as immunisations (*n* = 3) worked well in lower facility-birth-rate countries because parents usually take children for vaccinations even after home birth. eHealth innovations, where mobile phone technology was used by a registrar to register births within facilities, were used in two studies. Figure 2 outlines the facility-based birth registration interventions for the articles included in the review.

**Figure 3.**
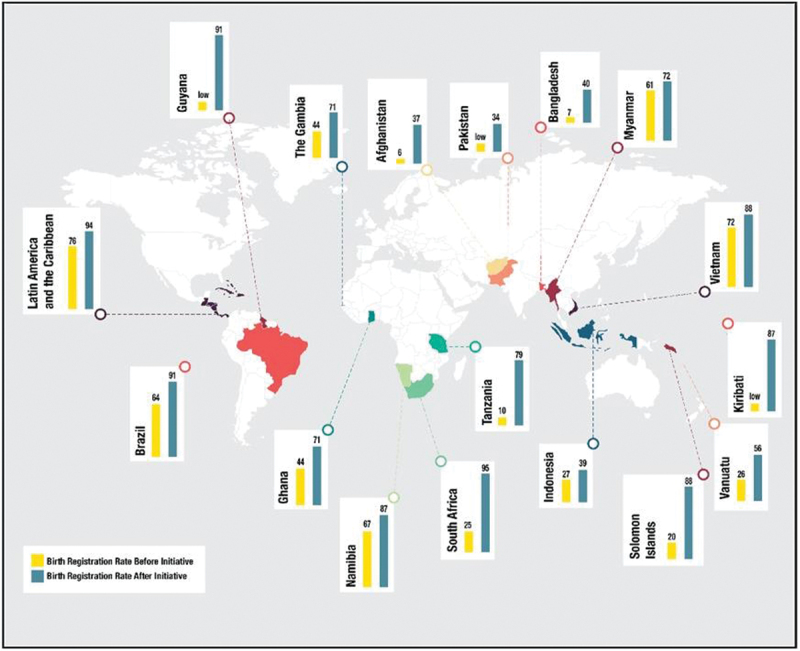
Global map displaying the birth registration rates before and after initiatives as found in literature review.

Birth registration rates were reported as a percentage of either under one years (*n* = 2), under five years (*n* = 1), or unspecified (*n* = 18). In all the papers (*n* = 21), birth registration rates increased after implementation of any of the interventions. More than one intervention was used simultaneously in many of the papers (*n* = 12). Solomon Islands, South Africa and Tanzania had the highest rate difference, increasing by 68%, 70% and 69%, respectively [5,8,20]. All three countries implemented decentralised birth registration initiatives by setting up CRVS offices within health facilities. Figure 3 presents the difference in birth registration rates before and after the interventions.

#### Barriers to facility-based birth registration

The articles described health systems-related, legal, and societal barriers to universal birth registration (see Table 1) [5,8,20–27]. Most commonly cited were lack of knowledge and financial cost, which refers to both demand and supply barriers. Financial cost arose as both a legal barrier, where cost of birth registration was required by law, and as a societal barrier, where there was an opportunity cost associated with registering the child at birth.

**Table 1.** Barriers to birth registration identified in the literature review articles (*n* = 21).

Barrier to Birth Registration	Number of articles mentioned (n)	Example of barrier cited from included articles
**Health Systems**		
Manual system	3	‘Efficiency of the current system is impaired by several factors including the salient fact that the system is almost entirely manual and highly centralized.’(26)
Geographic isolation	3	‘The lack of access to official facilities to register a child’s birth, especially in remote and rural areas as well as poorer regions, needed to be addressed. There was inadequate infrastructure in place to reach those who are hard to reach.’(20)
**Legal**		
Stringent laws	5	‘Within the current legislation, there are a number of areas highlighted as inhibiting birth registration in Vanuatu. The law states that all registrations need to be handwritten, multiple paper copies are to be produced and kept on file. This has resulted in cumbersome, largely paper-based system, prone to human error and unsuited to the geographical and technical barriers in Vanuatu.’(22)
Financial cost	9	‘ … financial barriers also inhibited registration rates as many families could not afford the cost of registration or the required transportation fees to access a registration post.’(19)
**Societal**		
Lack of knowledge	8	‘ … low birth registration rates are directly linked to the parents’ awareness levels about the range of benefits’(22)
Opportunity cost	2	barriers to birth registration, such as transportation costs and other opportunity costs incurred when families had to separately register their child’s birth at a Department of Home Affairs (DHA) office”(20)
Distance to travel	4	‘More than half of all children in Tanzania are born at home rather than a hospital or health facility. With only one Registrar office per district, many new parents must make at least two long journeys in order to register their child and collect a birth certificate.’(5)
Cultural reasons	3	‘Cultural practices such as the naming of a child at home and not in a health facility can delay timely registration of births.’(21)
Area of conflict	1	‘earlier birth registration system had fallen into disuse during the succession of conflict’(25)

### Semi-structured interviews with birth registration stakeholders

A total of six semi-structured in-depth interviews were conducted by MP (See Table 2 for interview log). Among 19 stakeholders initially contacted, two referred colleagues who agreed to an interview, 10 were unavailable or unresponsive to emails, and one stakeholder refused consent so was not interviewed.

**Table 2.** Key stakeholder interview log.

No	Date of interview	Type of Organisation	Interview method	Duration of interview
1	20/07/2018	NGO	Skype audio call	32 min
2	21/07/2018	NGO (retired)	Skype audio call	45 min
3	01/08/2018	Foundation	Skype audio call	23 min
4	02/08/2018	Foundation	Skype audio call	21 min
5	10/08/2018	NGO/University	Skype audio call	20 min
6	17/08/2018	International Agency	Skype audio call	42 min

#### Facility-based birth registration initiatives

Incorporating birth registration and CRVS into the health sector as a form of intersectoral collaboration was the most common theme identified among the participants.

Most of the stakeholders (*n* = 5) mentioned registration offices opening within healthcare facilities, including primary health facilities and larger hospitals. Such initiatives were reported to improve birth registration rates for the region and countries. One stakeholder described their work:

We work in 4 countries in the Americas getting the countries to open [CRVS] offices in hospitals: in Guatemala, Honduras, El Salvador and Paraguay… Our projects in this regard started in 2009, the last one ended in 2014 and the offices are still open and they’re registering the births – Stakeholder 1

Half of the participants spoke about having registrars work from health clinics as an outreach exercise to register children within the primary healthcare setting. One stakeholder described how this is currently working in Senegal:

We did what are called birth registration corners: a registrar be placed in the primary healthcare clinic on child days, and so they sit there and actually do registration, and then they can bring the birth certificates to give the moms. – Stakeholder 6

A different model was also described by some, where hospital staff, such as doctors or the hospital Chief Executive Officer, are given the power to act as registrar. This fully integrates CRVS with health sectors by giving health facilities the full responsibility and ability to register children at birth. It allows mothers the opportunity to have their births registered, and in some cases issued with a certificate before leaving the healthcare facility after delivery.

Another initiative that was mentioned by two stakeholders was to incorporate birth registration into immunisation programmes.

The main limitation about facility-based initiatives, as mentioned by all participants, is that the most vulnerable and marginalised groups, with less access to facility birth, are excluded. However, four of the respondents stated that having facility-based registration as an initial starting point is good. As one stakeholder said:

We’re currently running a campaign overall and I think when running a campaign, it’s important to pick quick wins, and obvious quick wins [are] if you’re having more births in facilities and hospitals would be to focus the campaign on that and close that gap. – Stakeholder 5

#### Barriers to facility-based birth registration

As informed by the literature review, we divided barriers to birth registration into three main themes: legal and governmental barriers, health systems barriers, and societal barriers.

*Legal and governmental barriers*: Four of the six stakeholders mentioned legal frameworks being a barrier, the fact that the law puts the onus on the parents rather than the health facility or civil registry to register the child.

In some countries the ministries involved in birth registration, statistics, or finance, do not collaborate well with the ministry of health, and there are laws in place which prevent hospitals from becoming registration centres.

One stakeholder explains the registration process in some countries, where the law results in an extra burden on parents to get their child registered:

… for example, in Rwanda and Burundi you need to register with the notification from the hospital, but you also need to bring 2 witnesses. – Stakeholder 2

Other legal barriers include stringent requirements needed at the time of birth registration which may not be available, including the child’s name (often not decided immediately after birth), the father’s name or a marriage certificate. Two respondents concluded that these legal barriers are a direct result of laws relating to birth registration being outdated, too stringent and requiring revision.

*Health systems barriers*: Most of the respondents described barriers within the health system itself including hospital staff being too busy to register children (even if they are empowered), poor infrastructure within hospitals to set up a registration centre, and the potential lack of security of the information. As one participant said:

… another burden on health resources in low- and middle-income countries [is that] I think they’re already stretched; the staff already work extra so this is an added burden on them. I think that’s another major disadvantage. – Stakeholder 4

Another stakeholder described the problem within both health and civil registry systems: staff are overworked and under-resourced.

*Societal barriers*: All participants stated that financial cost was a major barrier preventing children from being registered. This includes cost of travelling to birth registration centres and direct cost involved with registering the birth of a child.

Five out of the six stakeholders spoke about lack of knowledge regarding birth registration, both the benefits of birth registration and the process (where or how parents should register their child after birth). One participant explains this with regional context:

In other areas it’s just not understanding why registration is important and this happens in rural areas mostly and indigenous communities, for example in Latin America, where they don’t understand why it’s important to register a birth. – Stakeholder 1

Additionally, interviewees mentioned that the full responsibility for registration is placed on the parents which becomes a barrier if the parents are not empowered or informed, or if other barriers prevent them from completing registration. The stakeholder interviews complemented and added depth to the literature review, with no notable divergent findings.

## Discussion

Our review identified 43 birth registration interventions across the 21 articles which were used in isolation or combination to increase birth registration rates at facility level. After synthesising the literature and stakeholder interviews, we noted enablers that would enhance birth registration, including inter-sectoral collaboration between health sector and civil registration ministries e.g. placing civil registration offices in health facilities or allowing medical doctors to act as registrars. This interoperability between health services and CRVS can benefit both health programmes and CRVS. Importantly, health promotion within communities also increased demand for birth registration.

### Facility-based birth registration models and approaches

We identified initiatives that had purely supply-side approaches, or demand side, or both. In terms of supply-side, the dominant theme that emerged was the benefit of integrating CRVS into health system delivery to aid birth registration of newborns. Primary healthcare facilities and hospitals are closer to communities and in smaller geographical units allowing for easier access than the usually more centralised civil registration centres [28]. Decentralisation could overcome barriers experienced by rural communities, such as financial cost, travel time and opportunity costs [29]. This collaborative effort is currently used in Namibia, Vanuatu, Gambia and South Africa and has shown an increase in birth registration rates [8,21,22,30]. In Uganda and Costa Rica, medical doctors are given the power to act as registrar and register children at birth, which has also shown an increase birth registration rates [30,31].

In terms of demand side, promotion and awareness is highlighted as key in both the literature review and interviews, emphasising the need for well-designed health promotion campaigns. In many countries, parents are unaware of the importance of birth registration and may realise later once they require proof of identity for their child [28]. Facility-based health promotion strategies at routine healthcare touch points such as antenatal and immunisation clinics could inform women and increase the demand for birth registration. Integrating birth registration with immunisations presents an additional opportunity to check and register births when carers bring newborns for their birth or six-week immunisations, such as in the successful initiative in Bangladesh [30].

Linkage of documentation, such as in South Africa and Burundi, where birth registration information is incorporated into compulsory documentation for mother and child, has shown to increase birth registration rates [18]. Introduction of these compulsory documentation could be a key enabler and improve CRVS data collection overall in countries with lower birth registration rates.

### Barriers and enablers to facility-based birth registration

Several LMICs still have laws in place that require revision, such as the requirement of the father’s name for birth registration, which acts as a barrier because the father may be unknown or absent from the child’s life. Some countries require a fee to be paid to register the birth of a child [28], which adds an extra financial burden to the parents. Outdated policy should be reviewed and revised towards removing barriers imposed by stringent laws and requirements associated with birth registration.

In both the literature and interview findings, health systems barriers primarily focused on the lack of human and infrastructural resources within the health facility that impede their ability to register births on site. Staff working in health facilities in LMICs are already overworked. Empowering them to register births too will add to the already heavy workload [29].

Finally, societal barriers were the largest group of barriers, with cost of birth registration being the predominant one. This includes financial costs, including money required for transport and fees associated with registration, and opportunity cost, where parents forego other activities, such as employment, to complete birth registration. Lack of access to information poses another societal barrier, which reduces demand for birth registration [7]. Again, health promotion campaigns may improve awareness on the value of birth registration so that the public can make more informed decisions.

**Figure 4.**
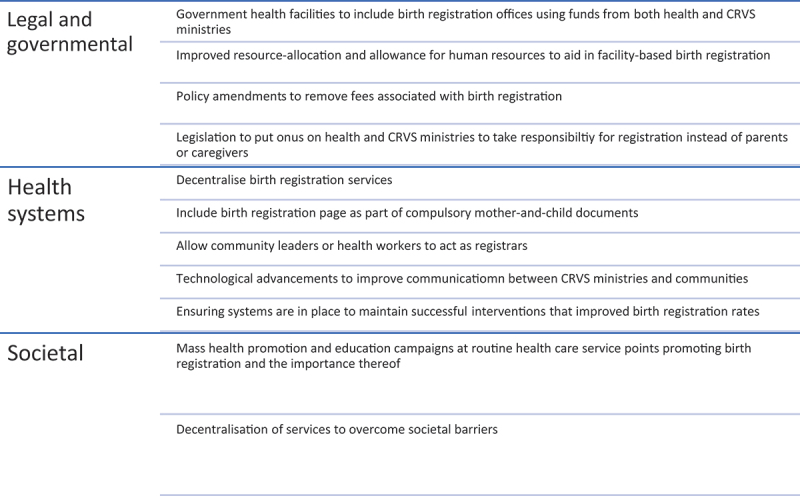
Recommendations for facility-based birth registration initiatives.

There have been prior reports and discussion papers on the linkage of CRVS and the health sector, and how this interoperability could improve outcomes [12,32–34]. The recent WHO Civil Registration and Vital Statistics Strategic Implementation Plan [35] is a result of this robust view of the health sector playing a pivotal role in CRVS. Where this research provides additional value is by combining a thorough literature review with experiences from key stakeholders in attempt to quantify and qualify the impact of facility-based birth registration initiatives in LMIC. Figure 4 summarises the recommendations we have concluded from this research.

### Strengths and limitations

Incorporating both peer-reviewed literature and grey literature was a key strength of the literature review. The bibliographic database search yielded only three papers that met inclusion criteria, and thus the grey literature added a more satisfactory yield of research and reports. The global coverage of results was also improved by including multiple countries and regions, which provided a wider breadth of information and allowed for more detailed analyses. However, the use of grey literature brought about some limitations. The grey literature focused on the work of NGOs, which might be biased due to funding or a desire to present only beneficial result or may result in over-reporting of results. Grey literature in general is difficult to critically appraise, and some of the peer-reviewed articles lacked information in the methodology sections (supplemental online material 2), so a formal assessment for bias was not done. The recommendations provided by the studies were context-specific and may not be generalizable to all LMICs. The reporting of birth registration rates were not standard across the literature in terms of time frame – some papers reported improved birth registration rates many years later, which could be influenced by confounding factors – and method of reporting – birth registration rates were reported as a percentage of either under one years, under five years, or unspecified, which limits the comparability of these results between the different regions. Further studies are required to enhance the validity of future reviews done on this topic.

The predetermined interview guide allowed for breadth of information to be gathered from the respondents. However, only six stakeholders were interviewed due to a low response rate. Further studies could include other stakeholders such as members from LMIC communities rather than only global stakeholders so that diverse perspectives from local levels could also be obtained. Additionally, due to the purposive non-random sampling, the generalizability of the results may be sub-optimal.

Further quantitative and qualitative research is required to provide data on effectiveness of initiatives and further refine recommendations of how varying contexts for CRVS can progress.

## Conclusion

There is a call by the WHO and UNICEF to improve birth registration rates globally – as is explicitly stated in the SDGs and evidenced by the surfeit of available grey literature on the topic. Birth registration has become an emerging priority for many countries, however, understanding regarding successful birth registration initiatives is lacking.

Ongoing health facility-based initiatives to improve birth registration rates in LMICs have been successful. SDG target 16.9 aims to achieve a legal identity for all globally by the year 2030. A paradigm shift is still needed regarding the importance of CRVS. Governments, organisations and foundations must work together to accelerate progress including supporting and implementing facility-based initiatives if registration of births are to improve CRVS to improve measurement for *Every Newborn*.

## Supplementary Material

Birth_Registration_Supplemental_online_material.docx

## Paper context

Many organisations are working within health facilities to improve birth registration rates, as it provides access to essential services. Data exists on the barriers to birth registration but not much is documented about these initiatives and the impact they have. This paper identifies birth registration initiatives and provides evidence on their impact to guide funders, organisations and government planning in order to achieve the global aim of a legal identity for all.
