# Supplementary material for: Closing the birth registration gap for Every newborn facility birth: literature review and qualitative research
Source: Glob Health Action. 2023 Dec 12;16(1):2286073. doi: 10.1080/16549716.2023.2286073 (PMC10795615; doi:10.1080/16549716.2023.2286073)
Supplement: Birth_Registration_Supplemental_online_material.docx [file ZGHA_A_2286073_SM0160.docx]

**Supplemental online material**

1. Search strategy
2. Data extraction tables
3. Stakeholder interview request letter
4. Interview guide protocol
5. Consent form
6. Codebook for broad themes

**Supplemental online material 1:**

**Search strategy**

**Academic articles**

PubMed:

((((((((("health facilities") OR "health facility") OR "healthcare facilities") OR "healthcare facility") OR hospital) OR hospitals) OR clinic) OR clinics)) AND ((((("birth registration") OR "birth certificate") OR "birth certificates") OR CRVS) OR ("civil registration and vital statistics"))

CINAHL Plus and Medline:

"birth registration" OR "birth certificate" OR "birth certificates" OR "CRVS" OR "civil registration and vital statistics" AND "health facilities" OR "health facility" OR "healthcare facility" OR “healthcare facilities" OR hospital OR hospitals OR clinic OR clinics

Global Health (OVID)

1. ("health facilities" or "health facility" or "healthcare facility" or "healthcare facilities OR hospital OR hospitals OR clinic OR clinics ")
2. ("birth registration" or "birth certificate" or "birth certificates" or "CRVS" or "civil registration and vital statistics")
3. 1 AND 2

Web of Science

1. TS=("birth registration" OR "birth certificate" OR "birth certificates" OR "CRVS" OR "civil registration and vital statistics") AND
2. TS=("health facilities" OR "health facility" OR "healthcare facility" OR “healthcare facilities" OR hospital OR hospitals OR clinic OR clinics)
3. #1 AND #2

Cochrane database

1. ("health facilities" or "health facility" or "healthcare facility" or "healthcare facilities OR hospital OR hospitals OR clinic OR clinics ")
2. ("birth registration" or "birth certificate" or "birth certificates" or "CRVS" or "civil registration and vital statistics")
3. #1 AND #2

**Grey literature**

Websites searched: Google, UNICEF website, WHO website, Plan International website.

Search terms used were similar for the academic literature but needed to be adapted for use on the websites. The terms included: “Birth registration”, "CRVS", "civil registration and vital statistics", “Facility”, “Hospital”, “healthcare facility”,

**Supplemental online material 2:**

**Data extraction tables**

Academic articles

**1.**

|  | **From the text** | **Page number** |
| --- | --- | --- |
| **General information** | | |
| Title | Effectiveness of the Maternal and Child Health handbook in Burundi for increasing notification of birth at health facilities and postnatal care uptake |  |
| Author | Kayo Kanekoa, Jacques Niyonkurub, Ndereye Jumac, Térence Mbonabucad, Keiko Osakie and Atsuko Aoyamaa |  |
| Publication type (report/journal article/abstract) | Journal article |  |
| Publication source | Global Health Action |  |
| Year | 2017 |  |
| Database | Pubmed/ EBSCO/ Global Health/ Web of Science |  |
|  |  |  |
| **Characteristics of included studies** | | |
| **Methods** | | |
| Aim of study | “To assess the effectiveness of the MCH handbook in increasing notification of birth ownership and PNC uptake in all 23 health facilities in the Gitega District, Burundi.” | 2 |
| Study design | “Pre- and post-introduction measurement were applied including: (I) structured interviews with two different sets of randomly selected mothers having infants aged less than six weeks at the pre- or post-studies and (ii) secondary data from the national health management information system.” | 2 |
| **Setting** | | |
| Country/Region of Focus | Gitega District, Burundi | 2 |
| Setting description | “Mountainous area of central Burundi about 100 km from the capital, Bujumbura” | 2 |
| **Participants** | | |
| Participants description | “2 administrative offices for birth registration and 23 health  facilities, that is, 2 referral hospitals, 1 clinic, and 20 health centres.”  “The estimated annual birth number in the Gitega District was 12,066, 67.4% (8136 births) of which occurred in health facilities” | 2 |
| Total number | 368 women from the 23 health facilities providing maternal and child health care services in the Gitega District | 2 |
| Demographic details | “Mothers with infants aged less than six weeks and living within the jurisdiction of each health facility.” | 2 |
| Sample selection | “Selected randomly from maternity registers, which are compiled at each facility.” | 2 |
| Sample size | “The sample size for the interview was calculated using the formula for single proportion” | 2 |
| Confounding variables | “Socio-demographic status”  “Delivery place by type of health facility” | 2 |
| **Intervention** | | |
| Details of intervention | Maternal and Child Health Handbook designed by a combination of stakeholders. Public announcement of the handbook was made prior to the pilot study. | 2 |
| **Data collection** | | |
| Details | “Structured interviews in November 2013 and July 2014 were conducted respectively with different sets of respondents with children of the same targeted age. Eight research assistants were trained for eight hours on study objectives, questionnaire contents, and interview techniques including ethical considerations. Researchers collected informed consent and conducted the pre- and post-studies with selected mothers by home visits. Completed questionnaires were checked every day by supervisors to ensure accuracy and completeness.” | 3 |
| **Outcome** | | |
| Outcome definition | 5 outcomes assessed:   1. Having MCH handbook 2. Having received the notification of birth at health facilities 3. Having data on delivery mode records at home 4. Having accurate birth weight data at home by recall-based or record 5. Receiving guidance on PNC from health personnel | 5 |
| Outcome of interest | “Proportion of mothers who received notification of birth at health facilities significantly increased from 4.6% to 61.0% (95% CI: 55.9%–66.2%).” | 4 |
| Responders | “Total valid responses obtained were 370 and 344 from the pre- and post-studies, respectively. There was no significant difference in the socio-demographic status of respondents who participated in the pre- and post-studies, in terms of age of mothers (p = 0.15), parity (p = 0.42), and final education level (p = 0.07)” | 4 |
| **Results** | | |
| **Theme 1: Barriers** | | |
| Barriers faced by population | Parents who fail to receive their children’s notification of birth at the health facility are required to take three steps for birth  registration: (i) to ask the village chief to make a notification  of birth attested by three witnesses; (ii) to declare the birth at an administration office using the previously obtained notification; and (iii) to obtain a birth certificate from the administration office. | 6 |
| **Theme 2: Facility BR initiatives** | | |
| Description of initiative | “pilot implementation of the Burundian MCH handbook, which contains  a page for notification of birth” | 2 |
| Strengths | “MCH handbook enabled clients to keep their own continuum records, which are essential for effective guidance of care.”  “ANC and delivery records inside the handbook could work as guides and references for care afterwards, even for use in maternal and neonatal death surveillance and response”  “birth weight data kept by mothers in the handbook would be vital to accurate child growth monitoring.” |  |
| Limitations | “Outcome variables were influenced by type of health facility where deliveries were taken”  “In particular, mothers who gave birth at hospitals/clinics had 2.62 higher odds (95% CI: 1.63–4.22) of obtaining notification of birth, and 3.25 higher odds (95% CI: 1.96–5.39) of having delivery mode records than mothers who gave birth at health centers. Conversely, mothers who delivered at hospitals/ clinics had 0.51 lower odds (95% CI: 0.51–0.81) of receiving guidance on PNC than mothers who delivered at health centers” | 5 and 6 |
| **Theme 3: Recommendations** | | |
| Key conclusions | The MCH handbook appeared to help health personnel provide guidance on PNC, thereby it may have increased the uptake. Furthermore, the MCH handbook ensured that parents possess their child’s notification of birth at a health  facility to induce the child’s birth registration with a simpler procedure. Recommendation to include birth registration into MCH handbooks throughout the country to improve the rates of BR. Also to increase health promotion during ANC. | 6 |

**2.**

|  | **From the text** | **Page number** |
| --- | --- | --- |
| **General information** | | |
| Title | Strengthening of local vital events registration: lessons learnt  from a voluntary sector initiative in a district in southern India |  |
| Author | Prem Mony,a Kiruba Sankar,a Tinku Thomasa & Mario Vaza |  |
| Publication type (report/journal article/abstract) | Journal article |  |
| Publication source | Bulletin of the World Health Organization |  |
| Year | 2011 |  |
| Database | EBSCO |  |
| **Characteristics of included studies** | | |
| **Methods** | | |
| Aim of study | “Objective of achieving target rates of 75% for birth registration and 50% for death registration.” | 379 |
| Study design | Not well described |  |
| Duration of intervention | 12 months (September 2007–August 2008) |  |
| **Setting** | | |
| Country/Region of Focus | Palamaner, Gangavaram, Baireddypalle, V Kota and Ramakuppam (Chittoor district of Andhra Pradesh state) in India | 379 |
| Setting description | “The programme was held in five *mandals* (subdistricts) – Palamaner, Gangavaram, Baireddypalle, V Kota and Ramakuppam– with a total population of 281 500 (in the year 2007) in Chittoor district of Andhra Pradesh state”  “Estimated annual number of births and deaths were 5320 and 2055 respectively, based on crude birth and death rates of 18.9 and 7.3 per 1000 respectively” | 379 |
| **Participants** | | |
| Participants description | Not well described |  |
| Total number | Not well described |  |
| Demographic details | Not well described |  |
| Sample selection | Not well described |  |
| Sample size | Not well described |  |
| Confounding variables | “It is possible that those who did not receive the household intervention were a biased group” | 381 |
| **Intervention** | | |
| Details of intervention | “Strengthening of Local Vital Event Registration (SOLVER) by a nongovernmental organization that worked with the government and the public at a subdistrict level in southern India.   1. Supply-side intervention: Awareness workshops on registration procedures within local government departments (education, health, social welfare). Arrangements made with hospitals for transmission on birth and death data to the government. 2. Demand-side interventions: Awareness within the community raised using media such as pamphlets, audio announcements and brief jingles on local cable television. Project staff visited households informing them of the benefits of vital event registration” | 379 |
| **Data collection** | | |
| Details | Not well described |  |
| **Outcome** | | |
| Outcome definition | “Improvement in birth and death registration rates | 381 |
| Outcome of interest | “80% of births and 61% of deaths were registered, as compared with pre-intervention rates of 50% of births and 25% of deaths.”  “Birth registration rose from 50% to over 80% in about 4 months after the start of the programme” |  |
| **Results** | | |
| **Theme 1: Barriers** | | |
| Barriers faced by population | “Political, administrative, economic and legislative barriers, and neglect of cultural and community realities act as constraints to the complete, accurate and timely registration of births and deaths in India.” | 379 |
| **Theme 2: Facility BR initiatives** | | |
| Description of initiative | “Strengthening of Local Vital Event Registration (SOLVER) by a nongovernmental organization that worked with the government and the public at a subdistrict level in southern India.   1. Supply-side intervention: Awareness workshops on registration procedures within local government departments (education, health, social welfare). Arrangements made with hospitals for transmission on birth and death data to the government.   Demand-side interventions: Awareness within the community raised using media such as pamphlets, audio announcements and brief jingles on local cable television. Project staff visited households informing them of the benefits of vital event registration” | 379 |
| Limitations | “Difficulty determining the contribution each intervention made towards improving registration, as well as uncertainty about sustaining registration at high levels in the long-term”  “those who did not receive the household intervention were a biased group”  “The focus and intensity of the intervention itself could have caused favourable outcomes (Hawthorne effect or observation bias).” | 380 |
| **Theme 3: Recommendations** | | |
|  | “Enable targeted interventions that are sustainable and cost-effective. For example, community campaigns, strengthening collaboration between government departments, capacity building of health staff, holding intensive mobile registration drives to target priority areas such as rural home deliveries and deaths of children and elderly people.”  “Further strengthening is needed on the evidence base on the social determinants of health and what works to improve them” | 381 |

**3.**

|  | From the text | Page number |
| --- | --- | --- |
| **General information** | | |
| Title | Impact of Policy Initiatives on Civil Registration  System in Haryana |  |
| Author | Pravin Kumar Singh, Manmeet Kaur, Nidhi Jaswal, Rajesh Kumar |  |
| Publication type (report/journal article/abstract) | Journal article |  |
| Publication source | Indian Journal of Community Medicine |  |
| Year | 2012 |  |
| Database | Pubmed |  |
| **Characteristics of included studies** | | |
| **Methods** | | |
| Aim of study | “Review of CRS system was conducted to study the process of policy change and the impact it had on the registration of vital events in Haryana” | 123 |
| Study design | Not mentioned |  |
| **Setting** | | |
| Country/Region of Focus | Haryana, India |  |
| Setting description | Not described |  |
| **Participants** | | |
| Participants description | Not described |  |
| Total number | Not described |  |
| Demographic details | Not described |  |
| Sample selection | Not described |  |
| Sample size | Not described |  |
| Confounding variables | Not described |  |
| **Intervention** | | |
| Details of intervention | “Haryana government revamped the registration system on 1st January, 2005. To ensure better implementation of various provisions of RBD Act, it was decided to get the work of registration done through Primary Health Centres (PHC) instead of Police Stations. This policy change was also meant to increase the number of registration centres so as to make registration more accessible to people.”  “Medical Officer-in-Charge of the PHCs were designated as “Registrar (Births and Deaths) vide Government Notification (3/49/97-3 HB-III dated 10 December 2004). Pharmacist of PHCs were declared as Sub-Registrar (Births and Deaths) under Section 7(5) of the RBD Act to assist the Registrar in performing day to day activities related to birth and death registration. Sub-registration centers were also started in five Government Hospitals so that free extract of birth or death registration could be handed over to the family/relatives at the time of discharge” | 123 |
| **Data collection** | | |
| Details | No details given |  |
| **Outcome** | | |
| Outcome definition | 1. Make the registration of births and deaths available at the doorstep 2. Allow PHCs to issue birth or death certificates | 122 |
| Outcome of interest | - In the rural areas of Haryana, the numbers of registration centers have increased from 175 to 413*.* - Birth registration was stagnant at around 70% till 2004, which has gone up to 95% during 2009 | 123 |
| Responders | No details given |  |
| **Results** | | |
| **Theme 1: Barriers** | | |
| Barriers faced by population | Cost of travel  Distance to CRVS centres |  |
| **Theme 2: Facility BR initiatives** | | |
| Description of initiative | “Medical Officer-in-Charge of the PHCs were designated as Registrar and Pharmacist of PHCs were declared as Sub-Registrar” | 123 |
| Strengths | Improvement in BR levels |  |
| Limitations | “Officials and health workers at various levels were initially hesitant to take up the registration task, though it was part of their routine duties to record births and deaths. PHC officials considered registration work as “difficult” since they did not have experience or training in handling legal matters related to registration of vital events. They did not perceive it as “their” work. Legal sensitivities involved were not appreciated in the beginning, i.e., Medical Officers who were designated as “Registrar” did not pay required attention to the tasks related to registration of vital events. Health workers too initially resented the work related to birth and death registration as it was considered as an “additional burden.” They felt that already they were overburdened with record keeping. Poor record keeping was seen in some registration centers such as cutting/overwriting in the records. Potential for misuse of loose blank forms that were easily available with the Anganwadi workers and Auxiliary nurse midwives was also observed initially” | 123 |
| **Theme 3: Recommendations** | | |
|  | “Periodic training and reorientation new system stabilized within a year. Periodic review at the state level and a word of caution by state Registrar kept the district authorities alert | 124 |
| Key conclusions | “It is possible to change policies that lead to improvement in the Public Systems; however, it is important to understand the process of policy change” | 124 |

Grey Literature

|  | General  (Title, Author, Publication, Year) | Stakeholders involved | Methods (study design, country, target population) | Theme 1: Barriers | Theme 2: Facility BR initiative | Theme 3: Recommendations | BR rate before | BR rate after |
| --- | --- | --- | --- | --- | --- | --- | --- | --- |
| 1. Guyana 2011 | - Towards Universal Birth Registration for Guyana: Report of Assessment Legislation, Policy and Practice on Birth Registration - UNICEF - Policy report - 2011 - UNICEF website | Rights of the Child Commission, UNICEF | Determine BR rates (quant) and KAP study (quali) on BR  Region: Guyana  POLICY REPORT | 1. Distance from registration centres 2. Fee for birth registration 3. Manual system, need for computerized system | “Guyana observes several of the approaches outlined in UNICEF‟s Best Practice Working Paper for birth registration such as the placement of registrars within health institutions and the engagement of community health officers as outreach registrars. “ | - “Redo the Registration of Births and Deaths Act and reflect the role of ministry of health and Ministry of Amerindian Affairs in the law” - BR should be free - Increase awareness, greater attention to eeds of the key groups involved (mothers, registrars, regional officials etc) - Plan for targeting children in remote areas |  | 91% |
| 2. Namibia 2015 | A Namibia Fit for Children: Birth Registration  UNICEF  2015  UNICEF website | UNICEF | Fact sheet about facility-based BR and effect on BR rates in Nambia from implementation in 2008 until 2010  REPORT | “Cultural practices such as the naming of a child at home and not in a health facility can delay timely registration of births. Late registration – the registration of children over the age of 1 – remains a concern. In 2012/2013, 57 per cent of all registrations were late registrations.”  ”Other challenges in birth registration relate to abandoned or orphaned children. Registration for such categories of children can be difficult, even if there is proof of birth, because the details of at least one parent are required”  “lack of awareness among parents of the value of birth registration to access social services and of the supporting documents needed for registration.” | “The Ministry of Home Affairs and Immigration, with UNICEF support, is increasing access to birth registration through mobile campaigns in remote areas and through hospital-based registration. Given that the vast majority of babies are born in health facilities, a hospital-based registration system was piloted with UNICEF support and subsequently expanded to other facilities. Today, registration is available in 22 hospitals with high numbers of births” | “UNICEF is working with partners to scale up access to birth registration using innovative and strategic solutions in the following areas:  • Using laptops with secure Internet lines (VPN) that can access the national population registration system remotely; this could facilitate verification to confirm identity of parents and help reduce late registrations,  • Strengthening birth registration in lower density hospitals/clinics through outreach upon a birth notified by the clinic, or a home birth notified by the traditional leader,  • Employing cell phone technology to transmit birth notifications to a regional or sub-regional office from remote locations. This would also facilitate some cultural renaming practices, while ensuring there is still the proof of birth required for registration,  • Expanding one-stop approaches to link the registration of birth with registration for a social grant, for example, by offering grant registration services in hospital or civil registration offices and conducting joint outreach on birth and social assistance registration by the Ministries of Health, Gender Equality and Child Welfare and Home Affairs,  • Implementing a communications campaign and social change interventions to address traditional practices and cultural barriers to timely registration.” | 31% (2008)  [Children under the age of one] | 55% (2010) |
| 3. Namibia | Ministry of Home Affairs and Immigration  GOVERNMENT OF THE REPUBLIC OF NAMIBIA  Operational Framework  for 2012-2013 | UNICEF  Govt of Namibia | Operational framework and report  Namibia  REPORT | As above | “Since 2008, UNICEF has supported the opening of 21 (out of a possible 34) hospital-based offices across the country. Most of these offices are in areas with low BR rates. Of the 21 offices, 19 are fully functional at the time of writing. UNICEF has also provided technical and financial support to 18 newly established sub-regional MHAI offices in areas where it is not cost-effective to set up separate hospital-based offices. The number of children registered at the hospital-based offices has increased steadily since 2008, with 23 575 registered in 2010. Data analysis confirms that since the introduction of the hospital-based offices, births are being registered earlier. So far, 9 of the 21 offices also register deaths”  “Responding to the challenge of low BR rates at many hospital-based facilities, the MHAI consulted with management of the Ministry of Health and Social Services (MoHSS) and regional ANC and PNC staff, where after information on BR (i.e. ‘why’, ‘where’ and ‘how’) was incorporated into ANC and PNC sessions” | Need to prevent late registration  Need to address low budget allocation | Under 5 BR 67.4% in 2006 | 75% in 2010 |
| 4. | Children and the  Namibian budget:  Social assistance  and welfare | UNICEF | Briefing papers  Namibia  BRIEFING PAPER | “father has to agree that his name appears on the certificate, and in some traditions the father-in-law has to provide the name for the child, which can take time.” | As above | As above |  | 87.1% in 2013 |
| 5. Vanuatu 2014 | Child protection case study, Counting Every Child, Partnerships Lifting Birth Registration Numbers in Vanuatu  UNICEF  2014  UNICEF website | Ministry of Education, Ministry of Health and UNICEF | Vanuatu  CASE STUDY | “1. The difficulty of capturing births in communities outside of hospitals and health centres (including kastom villages),  2. A high reliance on family to transfer data between departments  3. The need to improve coverage and accessibility to registration  4. Cost involved in travelling to a registration centre  5. Cost of registration.  6. Law: All registrations need to be handwritten, multiple copies to be kept on file; does not allow for those outside the Civil Registry to do birth registration; gaps in requirements for documentation of birth (eg traditional birth attendants do not need to keep a record); prohibits mother from registering the child without fathers name  7. low birth registration rates are directly linked to the parents’ awareness levels about the range of benefits” | “1. Establishing multi-sectoral partnerships and mechanisms: MOU signed between Ministry of Internal Affairs, the Department of Civil Registry, the Ministry of Education and the Ministry of Health.  Permanent placing of a civil registry office at the central hospital in the capital, Port Vila. Mother who give birth I hospitals can now register their baby’s birth and receive a birth certificate and photo while still in hospital.  Education ministry not allowed to enrol children unless they’re registered  Civil Registry sends people to assist by working in registration in the centres  2. Decentralising birth registration to provinces  Expanding provincial network to provincial health facilities  3. Database  With information required by government  4. Addressing socio-cultural attitudes and beliefs  Awareness campaigns to change attitudes and beliefs about BR” | - “Strengthen the National and Provincial CRVS Task Forces - Legislative law - improve - Strengthen the registration system - Continue efforts to decentralise system - Advocacy and education efforts” | 26% in 2008 | 56% in 2014 |
| 6. Case studies 2000-2009 | UNICEF GOOD PRACTICES IN INTEGRATING BIRTH REGISTRATION INTO HEALTH SYSTEMS (2000-2009)  CASE STUDIES: BANGLADESH, BRAZIL, THE GAMBIA AND DELHI, INDIA  Jan 2010  UNICEF  UNICEF website | UNICEF | Bangladesh  Brazil  The Gambia  Delhi, India  WORKING PAPER | Review of the literature  Primary data collection through interviews and email communications |  | “Include birth registration in health information systems and create demand for birth registration data within the MoH. Conduct advocacy with the Ministry of Justice to reform national laws and regulations to facilitate birth registration. Create mandates within the MoH for health professionals and traditional birth attendants to register births and deaths. Engage community health officers and midwives as outreach registrars. Include birth registration in public health campaigns. Establish a monitoring system, led by the MoH and civil registration authority, to ensure the continuous operation of the registration system – for example, by ensuring timely replenishment of registration supplies and registrar books.”  “Community health workers are best suited to undertake the dual responsibility of registering/certifying children and providing health care since  Include birth registration in health information systems and create demand for birth registration data within the Ministry of Health.  Conduct advocacy with the Ministry of Justice to reform national laws and regulations to facilitate birth registration  Engage community health workers as outreach registrars  Include birth registration in public health campaigns” |  |  |
|  |  |  | The Gambia |  | “Integrating BR into reproductive and child health services  Gambia empowers public health officers to act as deputy registrars, in accordance with the 1968 BDMR Act.  Linkage of BR to growth monitoring cards” |  | 32% in 2000 | 55% in 2005 |
|  |  |  | Brazil |  | “In addition to the monetary incentives granted to hospitals per child registered, UNICEF supported the implementation of the 2001 National Programme for the Promotion of Birth Registration, consisting of placing outreach units of the notary public within maternity wards in the states with lowest birth registration rates”  “Issues: high cost of maintaining a full functioning civil registration unit within a maternity ward, considering the low number of children born daily, is one of the main factors behind lack of sustainability of this approach.”  “10-step training tool developed to integrate CR into maternity wards” |  | 70% in 1991 | 88% in 2007 |
|  |  |  | Bangladesh |  | integrating birth registration with an immunisation programme | “Legal reform was essential to improving birth registration rates.  The Government needs to partner with civil society to improve birth registration rates. A network comprising government and non-governmental partners was established to promote birth registration” | 7% in 1996 | 40% in 2008 |
| 7. Brazil 2012 | Case studies on UNICEF programming in Child Protection  Birth registration in Brazil  UNICEF website | UNICEF  Brazil govt | Brazil  CASE STUDY | “Reasons for low birth registration rates include geographic isolation, lack of awareness of the benefits of registration and discrimination against minorities. Registration of children with indigenous names is commonly denied, although this practice is against the law. National legislation does not require registration of indigenous children, but not having a birth certificate may limit their ability to claim other rights. In addition, birth certificates are sometimes not required within areas reserved for indigenous populations, which reduces the incentive to register.” | “Legislation: In 1997, the Government amended laws to eliminate fees for birth registration”  “National Programme for the Promotion of Birth Registration: Monetary incentives were not enough to encourage maternity wards to take on birth registration. As a result, in 2001 the Government established the National Programme for the Promotion of Birth Registration, in collaboration with civil society organizations, corporations and the media. As part of this campaign, UNICEF has supported the deployment of civil registration services in public hospitals”  “Linkage with the Baby-Friendly Hospital Initiative: Directives issued in 2004 and 2008 regulating the Baby-Friendly Hospital Initiative require that at least 70 per cent of newborns leave the hospital with a birth certificate in order for the hospital to be certified baby-friendly.” | - “UNICEF is supporting expansion of the online registration system piloted in Pernambuco state to all 217 hospitals in the state and its introduction in other states.” - “Improve the national rate of registration to 95 per cent by 2013” - “Register all children under 5 years old by 2014” - “Broaden access to birth registration among excluded population groups in partnership with the Government and the National Indigenous People’s Foundation” - “Establish online registration units in hospitals with more than 300 deliveries each year, at a rate of 250 units per year through 2014” | 64% in 1998 | 91% in 2010 |
| 8. Pakistan 2013-2015 | PROGRESS REPORT 2013-2015  Results for children  in Pakistan  UNICEF  UNICEF website  July 2015 | UNICEF | Pakistan  Case study progress report  PROGRESS REPORT | “On the supply side, there are serious structural barriers affecting the majority of Union Council offices, such as a lack of capacity, lack of incentives for staff, and technological barriers.”  “On the demand side, many parents simply do not see any benefit in registering their child at birth. Poverty, illiteracy and lack of services in rural areas compound this fundamental barrier, and for many, the costs of registration – fees, cost of travel and time away from work – are simply too high to make it worthwhile” | “In June 2014, following the completion of a number of studies supporting the plausibility of applying mobile phone technology to increase new-born birth registration rates, UNICEF and mobile phone operator Telenor entered into a partnership with the Governments of Punjab and Sindh to utilize mobile technology to improve birth registration rates.”  “The “Telenor” approach identifies non-traditional “gate-keepers” – female health workers, marriage registrars and Telenor outlet staff – and trains them to record the fact of the birth of a child through completion of a birth registration application form using a web-based application on a mobile phone. Once completed, the form is transmitted directly to the respective Union Council Secretary to complete the registration process. To date, more than 30 Telenor outlet operators have been trained in Sindh.” | - Continue to use technology, it will likely help improve pap-based systems in a cost-effective manner - “UNICEF will continue to assist to develop a comprehensive CRVS strategy for Pakistan” |  | 34% in 2015 |
| 9. Pacific islands | FAIR START  FOR EVERY CHILD  UNICEF  2016  Case studies on 6 countries | UNICEF | Case study  Solomon Islands  Vanuatu  Kiribati  CASE STUDY | “Connectivity and geographical challenges (made up of numerous islands)  Lack of awareness to benefits of BR  Financial barrier – families cant afford BR or transport fees  Legal: Fathers name needs to be on birth certificate, laws outdated” | Established CRVS units within hospitals, decentralised CRVS  Developled e-registry system | Developing multi-sectoral partnerships is key  Raising awareness through culturally appropriate campaigns  Providing incentives for registration | 20% in 2007 (Solomon Islands)  26% Vanuatu  Kiribati: Lowest in pacific islands | 35430 births registered SI 2014  56% Vanuatu 2014  87% 2014 Kiribati |
| 10. Southern Asia | Improving  Children’s Lives  Transforming  the Future  2016  UNCIEF  UNICEF website | UNICEF | Case study, report on progress Afghanistan  PROGRESS REPORT | Issues during conflict resulted in poor system for birth registration | “Multisectorial approach with ministry of interior and ministry of health to integrate BR into health facilities and immunisation centres  Integrated ministry of religious affairs to do BR through mosques  Community elders to register births in the communities”  “Developed e-registry system with strict system and passwords” | “Continue with the current collaborations and initiatives  Free BR Raising awareness of BR to create demand  Combining BR with other services  Computerized BR system” | 6% in 2003 | 37% in 2011 |
| 11 South Africa | SItuatoin Analysis of Children in South Africa April 2009 | UNICEF  SA Govt | Situational analysis  South Africa  SITUATIONAL ANALYIS | “The main causes for under-registration of new births from the supply side lie in insufficient service  points; a limited range of services at points designated as full service points; under-utilisation of  alternative service points; poor communication; and prohibitive costs of service access for impoverished  clients.” | “In an effort to increase accessibility and enable more extensive current birth registration, a number of innovative collaborations between the Department of Home Affairs and other service providers have been introduced recently, such as 74 multipurpose community centres, 109 mobile units and 101 hospitals. These now make up 43% of all HA service points, but are currently under-utilised. The extension of birth registration services to selected hospitals facilitates immediate birth registration for newborn babies, preferably prior to discharge. Full uptake has not yet been achieved.” | “Concerted efforts of Department of Home Affairs, Department of Health and Local Government need to be speeded up to ensure that every child gets a birth certificate soon after birth and thus is able to exercise its rights of access to essential services. Communities need to be encouraged to acquire certificates or every child even if they have been born outside of a health institution.  Traditional leadership should also support this effort of documenting every child.” | 25% in 1998 | 72% in 2005 |
| 12 | REACHING THE  HARD TO REACH:  A Case Study of Birth Registration  in South Africa  November 2016  Prof Joseph Wong et al | Canada Research Chairs program; the Ralph and Roz Halbert Professorship of Innovation at the Munk School of Global Affairs; and the MasterCard Center for Inclusive Growth | Working paper  South aFRICA  WORKING PAPER | - “black South Africans were understandably reluctant to register their children’s birth for fear of the apartheid regime” - “The lack of access to official facilities to register a child’s birth, especially in remote and rural” - “inadequate infrastructure in place to reach those who are hard to reach” - “lack of awareness surrounding the importance of birth registration and legal identity for children” - “challenges of bureaucratic coordination resulted in inefficient processes for birth registrat” - “As a 2007 report from a South African NGO pointed out, in South Africa the birth registration process involved six different government ministries.” | “increase access to health facilities in rural areas in South Africa, with a specific focus on mother and  child health”;  “Beginning in 1996, the National Health Information System for South Africa (NHIS/SA) led an initiative to integrate the birth registration process into the health care delivery system by implementing registration points in health facilities”  “in 2004 an online birth registration scheme was piloted in three hospitals in KwaZulu-Natal in an effort to directly integrate the birth registration process with birth delivery. The online scheme has since been scaled to nearly 400 health facilities across the country”  “The NHIS/SA also trained health practitioners and other front-line professionals to assist new mothers in filling-out birth registration forms after their child is born. Antenatal programs have also been revamped to include information about the benefits, procedures and requirements of birth registration. Postnatal programs, such as the distribution of the Road To Health booklets and follow-up immunisation procedures, similarly highlight the importance of birth registration and encourage on-time registration. Integrating registration points and birth registration procedures within health facilities and the antenatal and postnatal programs has reduced previous barriers to birth registration, such as transportation costs and other opportunity costs incurred when families had to separately register their child’s birth at a Department of Home Affairs (DHA) office”  “increase awareness among both new parents and health professionals about birth registration and its benefits.  “actively reach those who are hard to reach, creating several mobile programs that essentially bring social services – including birth registration – to children and their families living far from urban centers” | - “Birth registration is an integral part of the broader health care system. Increased access to health facilities in South Africa, specifically for mothers and children, has increased birth registration” - Integrate services - Build in incentives (child support grand needs BR to be applied for) - Active reach |  | 95% in 2012  86% one year BR in 2014 |
| 13. Indonesia | Birth registration for all in Indonesia:  A Roadmap for  Cooperation | Plan International Indonesian Ministry of Home Affairs | Progress report  Indonesia  PROGRESS REPORT | Poorer households suffer more | “In May 2016 Minister for Home Affairs issues a Circular to all Provincial Governors and City Mayors and requests them to accelerate services for the recording and the publication of electronic ID cards and birth certificates.20 The Minister commences by indicating that at present only 86% of the population has an e-ID card and 61.6% a birth certificate. The Minister asks Heads of civil registration and population offices to collaborate with the Heads of Education, Health and Hospitals in the regions to actively take birth certificate services to schools (kindergarten, primary and secondary schools, vocational schools as well as hospitals, health and birth centres).  Also: removal of fees, and collaboration with other secotors including education” | - “Incorporate other sectors more like health immunisation visits or schools where schools check students birth certificates” - “Mobile services with the help of religious sector” | 27% in 2010 | 39% in 2015 |
| 14. Tanzania | Birth Registration in Tanzania: Tigo’s support of the new mobile birth registration system  2016  GSM Association | GSMA  UNICEF  UKAID | Progress report  Tanzania: Mbeyo  PROGRESS REPORT | “More than half of all children in Tanzania are born at home rather than a hospital or health facility. With only one Registrar office per district, many new parents must make at least two long journeys in order to register their child and collect a birth certificate. In areas that lack adequate infrastructure and public transportation, travelling this distance can be prohibitively time-consuming, expensive and inconvenient. Many parents simply lack the financial means to pay for the cost of travel and the mandatory registration fee.”  “lack of awareness about how to complete the registration process and the benefits that accompany registration are also obstacles” | “In 2011 RITA developed, with support from UNICEF and Tigo, a five-year birth registration strategy that aimed to make the process more affordable, widely accessible and efficient. The new strategy was initially piloted in one district of Dar Es Salaam (Temeke), before being scaled into two additional regions: Mbeya and Mwanza. As a first step, RITA eliminated the TSH 3,500 processing fee, making it possible for parents to register their child and obtain a hand-written birth certificate free of charge. The registration process was also decentralised; rather than requiring parents to register their child and request a certificate at the District Registrar office, local Registration Agents (or registrars) from local government administrative offices, hospitals and health clinics were trained to provide these services. This expanded the average number of registration touchpoints in each district from one location to forty and greatly reduced the maximum distance parents had to travel. With these changes,  it became possible for a parent to travel to a local health clinic to register their child’s birth, have their  child vaccinated, and have a handwritten birth certificate produced all in a single trip.” | “key aim for the initiative in the coming months will be embedding the new registration system into law,  and working together to make mobile registration sustainable for the government in the long-term” | Mbeya Tanzania  8% | 45% in 2016 |
| 15. Tanzania | Mapping Access to BR and Updates from Tanzania  Matthew Wilson  Digital Identity  September 2017  GSMA.com | GSMA  UNICEF  Govt of Tanzani | Website article  WEBSITE | As above | As above | As above | 10% in 2012 | 79% in 2017 |
| 16. Ghana | Bulletin of the WHO: Birth registration and access to health care: an assessment of Ghana’s campaign success  Bulletin of WHO  2013  Fagernas, S  Odame, J | UNICEF  Plan International  WHO | Bulletin | Fees  Indirect costs such as transport  Lack of awareness | “BR centres are placed within or nearby health facilities with registrars on site  Public education”  “Annual health promotion weeks y birth and death registry”  “Health services outreach registration” | “Incorporation of BR into health sercvies and health campaigns can increase BR”  “Need to strengthen the links between health services in BR”  “Actively encourage those in remote araas to register their children” | 44% in 2003 | 71% in 2008 |
| 17. Latin America and the Carribean | BIRTH REGISTRATION IN LATIN  AMERICA AND THE CARIBBEAN:  CLOSING THE GAPS  2016 Update | UNICEF | REPORT | Barriers more common in rural areas  Wealthier get registered | Establishment of BR services in hospitals | Focus on the more vulnerable and excluded groups | 76% in 2000 | 94% in 2016 |
| 18. Myanmar | Assessment of the Myanmar  Birth Registration System  Tomas Africa  UNICEF  2013 | UNICEF | Report  REPORT | “Penalty fees for late registrations  Strict laws unnecessary  Specific mention of religion and ethnicity on the ID card “ | “The Modified Vital Registration System is administered by the Central Statistical  Organization and Department of Health, which gives Township Medical Officers the task to issue birth certificates. Midwives are mandated to collect and transfer information about new born babies.” | “Improve birth certificate design to international standards  Lift restrictions on late registrations  Clarify the law better” | 60.6% in 2000 | 72.4% in 2013 |

**Supplemental online material 3**

**Stakeholder interview request letter**

Dear XXX

**Re: Closing the gap of uncounted children born in facilities globally**

I am contacting you as an expert in the field of data and Civil Registration and Vital Statistics (CRVS), specifically birth registration. I believe your input and knowledge would be invaluable to my masters project for the London School of Hygiene and Tropical Medicine (LSHTM) in collaboration with the United Nations International Children’s Emergency Fund (UNICEF).

I am a student at LSHTM doing an MSc in Public Health. For my dissertation, I am conducting qualitative research and a literature review on innovations to bridge the large gap between facility-based births and birth registration. The aim is to conduct in-depth interviews with key stakeholders to identify these gaps in order to provide recommendations on how facility-based birth registration rates could be improved in low and middle income countries (LMIC).

UNICEF estimates that around 230 million children under the age of five worldwide has not been registered at birth with the least developed countries having an average of 40% of births registered. Identity registration is a basic human right in itself and is crucial in providing children with access to healthcare and education. Approximately 99% of unregistered births occur in low or middle income countries with nearly 80% taking place in Southern Asia and Sub-Saharan Africa.

In order to obtain enough information for this project I will be conducting in-depth interviews with key stakeholders via Skype or phone call, each lasting approximately thirty minutes. The interviews will help inform my knowledge and understanding of facility-based birth registration initiatives and CRVS on a global and local level. I aim to use the data to identify gaps and provide recommendations for possible policy changes and future research.

I am writing to you to ask if you would please assist by taking part in an interview. I believe the insight gained from an interview with you or your team would broaden my knowledge and allow me to make a greater impact. All information obtained will be kept strictly confidential. Data collected will not be shared outside of the study team. The study is completely voluntary and you are entitled to withdraw at any point.

If you require further clarity or any other information, please find my contact details below. I look forward to hearing from you and hopefully arranging a convenient time for an interview this month.

Kind regards

XXX

MSc Public Health Candidate (LSHTM)

[XXX@student.lshtm.ac.uk](mailto:XXX@student.lshtm.ac.uk)

**Supplemental online material 4:**

**Interview Guide Protocol**

**Interview Guide Protocol**

**For experts in metrics/CRVS/facility-based birth registration initiatives**

**Review of birth registration initiatives and innovations in low and middle income countries targeting facility births**

*My name is XXX. I am a student at LSHTM doing an MSc in Public Health. For my dissertation, I am conducting qualitative research and a literature review on innovations to bridge the large gap between facility-based births and birth registration. The aim is to conduct in-depth interviews with experts in the field to identify these gaps in order to provide recommendations on how facility-based birth registration rates could be improved in low and middle income countries (LMIC) globally.*

*Thank you for agreeing to take part in this study. The interview should take approximately 30 minutes. Please read through this interview schedule at your convenience prior to the interview. As explained, you are free to stop the interview at any time or ask to skip a question should you not wish to answer. I want to also remind you that all your responses are strictly confidential. No identifying information of yours will be used in my report.*

**Introduction/Interviewee Information:**

Name:

Organisation:

Title:

1. **Professional background of respondent**

- What is your professional background?
- Please describe the work you have done of relevance to CRVS or birth registration.
  - *If LMIC not mentioned:*
    - What experience do you have working in low or middle income countries on CRVS and/or birth registration?

1. **Knowledge about birth registration in facilities and the gap**

- In LMIC currently, approximately 80% of births are delivered in facilities, but less than half are being registered at birth. This is large gap, why do you think this gap exists?
- In your experience, what are the main barriers to birth registration on a global scale?
- How is your organisation working to close this gap?
- What important initiatives and/or innovations are currently trying to improve birth registration rates overall on a global scale?

1. **Knowledge about initiatives and innovations for birth registration targeting facility births**

At the moment there is a move towards birth registration in facilities that the birth is taking place in.

- Why should we target birth registration in facilities instead of non-facility? Explain.

Earlier we discussed innovations targeting birth registration in general. I now want to focus specifically on facility-based birth registration initiatives.

- What innovations are there to improve *facility-based* birth registrations?
- What are three strengths to facility-based birth registration in low and middle income countries?
- Describe three limitations to facility-based birth registration in low and middle countries?
- How would you propose to bridge these gaps/overcome these? Do you think there are ways to improve the initiatives you mentioned?

1. **Achieving the SDGs**

*SDG 16.9 Sustainable Development Goal (SDG) number 16 is to “promote peaceful and inclusive societies for sustainable development, provide access to justice for all and build effective, accountable and inclusive institutions at all levels”. Goal 16.9 refers directly to birth registration with the aim to have legal identity for all globally by 2030.*

- Do you think we will be able to achieve SDG 16.9 by 2030?
- How do you think *facility-based* birth registration could contribute towards achieving SDG 16.9?
- Do you have other recommendations other than initiatives previously mentioned to help get closer to that goal of everyone being registered at birth by 2030?

*Thank you so much for taking the time to talk to me and take part in this study. I will use the information collected to produce a literature review and recommendations for submission to LSHTM by mid-September. The information gathered may also be used in reports, and possibly related peer-reviewed publications. If you have any questions at any point after this interview please contact me via email.*

**Supplemental online material 5:**

**Consent form**

**Information for Interview Participants**

Review of facility-based birth registration initiatives and innovations in low and middle income countries

*You are being invited to take part in a research study as a global/national expert. Choosing to take part is entirely at your discretion. Before you decide to participate, it is important for you to understand why the research is being done and what is required of you. Please read the following information carefully. If anything you read is unclear or if you would like more information please ask for further clarification.*

**Who is doing this study?**

I am a student at the London School of Hygiene and Tropical Medicine (LSHTM) doing an MSc in Public Health. The research for this study is in collaboration with the United Nations International Children’s Emergency Fund (UNICEF) and LSHTM as part of my dissertation for the MSc.

**What is the purpose of the study?**

The aim of this project is to identify innovations to help bridge the gap between facility births and low birth registration rates.

**What will happen to me if I take part?**

If you agree to take part in the study, you will be interviewed by myself for approximately thirty minutes on a day and time that suits you. The interview will be in person, via Skype or other similar means. The interview will be recorded and later transcribed by me, after which the recording will be permanently deleted.

**What are the possible benefits of taking part?**

The information I obtain from the interviews will help inform my knowledge and understanding of facility-based birth registration initiatives and CRVS on a global and local level in low and middle income countries. I aim to use the information to identify gaps and provide recommendations for possible policy change and further research in the future.

**Will my taking part in the study be kept confidential?**

Yes, it will. All identifying data will be removed, and identification numbers will be assigned to ensure complete anonymity. The data collected about you during the course of the study will be kept strictly confidential and not shared with anyone outside the study team. The data will be securely stored with password protection. If any quotes are used, they will be anonymized and you will not be identifiable to anyone.

**What will happen to the results of the study?**

The information gathered will be used in reports, and possibly related peer-reviewed publications.

**What will happen if I do not want to carry on with the study?**

The study is voluntary and you are entitled to withdraw at any point without giving any explanation about your decision. If you decide to leave the study, any data (whether it is a partial or complete collection of data) that refers specifically to you will be destroyed and researchers will not be allowed to use it in this study or in the future.

Thank you for taking the time to read this information.

**Participant Consent Form**

Participant study ID:

I confirm that I have read and understand the participant information sheet for the above study. I have had the opportunity to consider the information, ask questions and have had these answered fully.

I understand that my participation is voluntary and I am free to withdraw at any time.

I understand that sections of data collected during the study may be looked at by responsible individuals from the London School of Hygiene & Tropical Medicine, where it is relevant to my taking part in this research.

I agree to take part in the above study.

I agree for my anonymised quote(s) to be used in publications and reports.

| Name of Participant or Legal Guardian *(printed)* |  | Signature or thumb print impression of participant or legal guardian |  | Date |
| --- | --- | --- | --- | --- |
| Investigator: |  | Signature |  | Date |
|  | | | | |

Place: __________________________________________

Contact: XXX@student.lshtm.ac.uk

**Supplemental online material 6:**

**Codebook for broad themes on barriers to birth registration**

| **Code** | **Definition** |
| --- | --- |
| Health system | Any mention of factors related to healthcare facilities, providers, and procedures that may hinder birth registration. This includes issues like access to healthcare facilities, healthcare provider knowledge, medical record-keeping, and provision of health education. |
| Legal/governmental | Challenges associated with government policies, legal requirements, and administrative processes that can act as barriers to birth registration. This includes complex registration procedures, legal documentation, administrative delays, legal requirements, and costs/fees. |
| Societal | Social and cultural factors, as well as issues related to discrimination, poverty, migration, and awareness within communities, which can impact the willingness and ability of parents to register their child's birth. |
